# Analysis of factors predicting mortality of new patients commencing renal replacement therapy 10 years of follow-up

**DOI:** 10.1186/1471-2369-15-20

**Published:** 2014-01-20

**Authors:** Oliver T Browne, Victoria Allgar, Sunil Bhandari

**Affiliations:** 1 Hull York Medical School, Hull, East Yorkshire, UK; 2 Department of Renal Medicine, Hull and East Yorkshire Hospitals NHS Trust, Kingston upon Hull HU32JZ, UK; 3 Department of Statistics, Hull York Medical School and University of Hull Hull, UK

**Keywords:** Calcium, Chronic kidney disease, eGFR, Diabetes, Dialysis, Mortality, Phosphate risk factors, Vascular

## Abstract

**Background:**

The natural history of patients commencing dialysis in East Yorkshire is not well characterised and there is little convincing evidence which has studied the impact of potential factors prior to commencement of renal replacement therapy (RRT) at predicting mortality during dialysis. The aim of this study was to examine the previously published 5-year data on end stage renal disease and co-morbid risk factors for mortality at 10 years.

**Methods:**

An observational cohort study of subjects commencing dialysis in 2001/02 in East Yorkshire with a mean follow up from dialysis initiation of 8.8 years. Predictors of mortality were determined by univariate, multivariate analysis and survival via Kaplan-Meier analysis. Assessment of the utility of the Tangri risk calculator was carried out in addition to slope change in eGFR prior to dialysis commencement.

**Results:**

Baseline characteristics and the preferred mode of dialysis remained concordant with the original trial. The mortality rate at the end of the study period was 60% (56/94) with 30% (29) of patients having been transplanted. Highlighted in the 5 year data a significant proportion of mortality was made up of vascular disease and sepsis (71%) but this proportion had decreased (57%) by 10 years. Cardiac disease was the commonest cause of death but notably in 18% of patients, death was related to dialysis or withdrawal of treatment. Vascular disease and diabetes remained independent risk factors and predicative of mortality. Calcium - phosphate product which was associated in the early years with mortality was not in later years. Use of the risk calculator was predictive of commencement of RRT but not mortality but slope change in eGFR was predictive of mortality.

**Conclusions:**

Although diabetes and vascular disease remained predictive of mortality, interestingly calcium-phosphate levels are no longer significant and may be a more specific predictor of early cardiac mortality. Slope eGFR changes prior to RRT are a predictor of mortality. We speculate that aggressive management of cardiac risk factors in addition to early transplantation may be key to influencing the impact of survival in this cohort in addition to possible measures to delay renal progression.

## Background

Chronic kidney disease (CKD) has been increasingly recognized as a risk factor for mortality and cardiovascular disease. In the United Kingdom at least 7% of the adult population have an eGFR of less than 60 mls/min/1.73 m^2^ and over forty thousand patients are receiving treatment for end stage renal disease (ESRD) [1], with an increasingly aging population suffering from a significant number of co-morbidities these figures continue to increase at 5% per annum [2].

Despite advances in the treatment of chronic renal failure the prognosis remains poor. Timely identification of risk factors that predispose or exacerbate CKD will help to reduce the morbidity and mortality of those who will need future renal replacement therapy (RRT). In particular, there is strong evidence for cardiovascular risk factors being associated with deterioration in patients’ renal function and are predictive of those who will go on to require dialysis [3]. This generates scope for primary preventative interventions, ameliorating, not only classical modifiable risk factors such as; smoking, hypertension, diabetes mellitus and hyperlipidaemia but also non-traditional risk factors such as; functional iron deficiency, anaemia, insulin resistance and vitamin D deficiency [4-6]. Preventive measures are not only needed to prevent disease states occurring but also are required to modify outcomes once disease has been established. Secondary and tertiary preventative measures show that modifying a patient’s risk factors on dialysis therapy improves survival and quality of life [7-9]. In addition measures delaying renal progression, (eGFR being a strong surrogate cardiac risk factor), and hence cardiovascular risk may have a significant impact on future survival [10,11].

Previously we have described the 1 and 5-year results of an analysis of factors and co-morbidities predictive of mortality in a prospective study of a cohort of 94 dialysis patients [12]. Risk factors, which were analysed at 5 years, confirmed that an elevated calcium phosphate product, diabetes mellitus, vascular disease (such as peripheral vascular disease) and older age at the start of dialysis were independently predictive of mortality. In addition, the study identified low haemoglobin and lower eGFR prior to commencement of dialysis as good predictors of early mortality reflecting findings from other investigators [13,14]. We now present the extended ten-year data to highlight the impact and changes in risk factor profile and also further analysis using the Tangri risk calculator [15] and measures of slope change in eGFR prior to commencement of RRT.

## Methods

The design of the study was an observational cohort, taken from patients receiving dialysis from the Hull and East Yorkshire Hospital Services over a 10-year period. Re-analysis of the data was performed on the same cohort of 94 patients, the database from the 5-year period having been adjusted to include changes in patient outcomes. Previously analysed risk factors were sought for revalidation of predicting mortality.

The NHS Hospital Trust Research and Development committee approved the study as part of regional development of its dialysis service. All patients are globally consented for use of patient data prior to initiating dialysis with the service as set out by the Trust’s renal handbook policy. Formal written consent was not required as any subsequent interventions made during the observational study were aimed at improving clinical care based on the current guidelines, best available evidence and clinical expertise.

The 94 patients had been selected from the East Yorkshire region having commenced renal replacement therapy (RRT) in the form of haemodialysis or peritoneal dialysis in the year 2001/2002. All patients presenting for dialysis therapy were included except patients dialysing for less than 90 days, who were considered to be acute. Patient characteristics are described in the original study [12]. All cause mortality was the primary outcome measure and was used as a comparison to the previous 5-year data.

Factors analysed at the time of RRT commencement included; age at the start of dialysis, gender, smoking status, previous referral eGFR and eGFR at RRT initiation (measured by the four-variable Modification of Diet in Renal Disease equation), duration of renal care, renal disease aetiology, dialysis mode (haemodialysis and peritoneal dialysis), form of access, dialysis planning, diabetes and left ventricular hypertrophy (LVH) (diagnosed by ECG or trans-thoracic echocardiography).

Presence of significant co-morbidities at the start of RRT including; diabetes, vascular disease (defined from the presence of symptoms of peripheral vascular disease, clinical findings confirming vascular compromise, investigations confirming compromise – e.g. doppler, angiography or intervention with angioplasty or surgery (peripheral artery bypass grafts)), chronic obstructive pulmonary disease, ischaemic heart disease, cerebrovascular disease and visceral malignancy were studied together to stratify subjects into low-medium and high risk groups on mortality.

Modifiable biochemical markers of haemoglobin, serum albumin, triglycerides, cholesterol, calcium and phosphate at the start of RRT were also included in the analysis. Calcium-phosphate product was split into quartiles and the upper quartile was compared to the 3 lower quartiles when comparing effect on mortality.

Using the Tangri risk calculator (*a predictive model for progression of chronic kidney disease to renal failure)* the risk of entering end-stage renal failure (ERRF) was calculated in the cohort [15]. Calculations were made based on; age, sex, eGFR, calcium, phosphate, albumin and bicarbonate, before undertaking renal replacement therapy. The stratification in level of risk was: *0–10% = Low risk, 10–20% = intermediate risk and >20% risk of entering ESRF = High risk.*

The slope eGFRs for this cohort were calculated using linear regression from time of presentation to a nephrologist and just before commencing RRT. Those crash landing to dialysis within 90 days were censored from the analysis. The cohort was split into quartiles. The upper quartile (composed of the fastest decreasing slope) was compared against the lower three quartiles for mortality using Kaplan-Meier survival analysis.

### Statistical analysis

All continuous data are expressed as means (standard deviation (sd)), medians (interquartile ranges). All categorical data are expressed as number and percentage.

The first stage in the analysis was to describe the mortality rate at 10 years (Table 1). The independent variables were compared between those who were alive or dead at 10 years using a chi-square test tor continuous variables and chi-square tests for categorical variables (Table 2).

**Table 1:** **Mortality of study population over 10 years**^**a**^

**Total deaths over the 10 year study period including deaths in transplant patients:**		**65 (69%)**
	**Causes of death over 10 year period (56/94 = 60%)**^**a**^	**Causes of death over 5 year period (39/94 = 41%)**
Cardiovascular	17 (30%)	15 (39%)
Sepsis/infection	15 (27%)	13 (33%)
Malignancy	6 (11%)	5 (12%)
Dialysis related	3 (5%)	1 (3%)
Treatment withdrawn	7 (13%)	3 (8%)
Other	8 (14%)	2 (5%)

^a^ Deaths have been censored for live and deceased donor renal transplantation.

**Table 2:** Univariate comparison of Factors between patients alive and dead at 10 years

**Factor**	**Alive at 10 years**	**Dead at 10 years**	**P-value**	**Statistic**
Age (years) ^a^	53.2 (15.3)	69.0 (12.4)	<0.001	t(92) = 5.494
Vascular disease – no (%)^b^	25 (52%)	23 (48%)		
Vascular disease – yes (%)^b^	13 (28%)	33 (72%)	0.019	*X*^2^(1) = 5.535
Diabetes – no (%)^b^	29 (48%)	31 (52%)		
Diabetes – yes (%)^b^	9 (27%)	25 (73%)	0.038	*X*^2^(1) = 4.307
Co-morbidity (>3) –no (%)^b^	31 (60%)	21 (40%)		
Co-morbidity (>3) – yes (%)^b^	6 (15%)	35 (85%)	<0.001	*X*^2^(1) = 20..085
Haemoglobin >110 g/l (%)^b^	5 (50%)	5 (50%)	0.514	*X*^2^(1) = 0.426
Haemoglobin <110 g/l (%)^b^	33 (39%)	51 (61%)		
Creatinine at referral (μmol/l) ^a^	483.8 (± 77.9)	411.1 (± 28.6)	0.321	t(92) = 0.999
eGFR at referral (ml/min/1.73 m^2^) ^a^	18.6 (± 2.8)	19.3 (± 1.8)	0.838	t(92) = 0.205
Creatinine at start of RRT (μmol/l) ^a^	903.6 (± 59.9)	757.8 (± 27.3)	0.016	t(92) = 2.455
eGFR at start of RRT (ml/min/1.73 m^2^)	6.35 (± 0.4)	6.35 (± 0.2)	0.988	t(92) = 0.015
Albumin at start of RRT (g/L) ^a^	32.0 (± 4.9)	28.6 (± 5.7)	0.004	t(92) = 2.946
Haemoglobin at start of RRT (g/l) ^a^	95.6 (± 2.8)	87.8 (± 2.2)	0.026	t(92) = 2.264
Cholesterol at start of RRT (mmol/l) ^a^	5.2 (± 0.21)	5.0 (± 0.2)	0.685	t(92) = 0.406
Triglycerides at start of RRT (μmol/l) ^a^	2.12 (± 0.22)	2.19 (± 0.16)	0.821	t(92) = 0.227
Calcium phosphate product at start of RRT (mmol^2^/l^2^) ^a^	4.84 (± 0.29)	4.44 (± 0.22)	0.262	t(92) = 1.129

^a^ Age: analysis using *T*-test.

^b^ Chi-squared test.

For survival over 10 years based on time to death or last follow-up, the Kaplan-Meier method was used. Log rank tests were used to explore the relationship with categorical independent variables (e.g. presence of vascular disease, diabetes and age (less than or equal to 65 years vs greater than 65 years). This data is presented in Table 3.

**Table 3:** Kaplan-Meier survival analysis of factors affecting mortality

**Factor**	**Mean survival(Months)**	**95% CI**	**P-value (Log rank test –Mantel Cox)**
Age <65 years	85.4	(69.2-101.7)	
Age >65 years	51.7	(40.1-63.3)	0.001
Vascular disease	52.3	(38.6-66.0)	
No vascular disease	77.4	(63.891.0)	0.014
Highest quartile of the calcium phosphate product	60.4	(38.3-82.5)	
Lowest quartile of the calcium phosphate product	65.0	(54.8-77.2)	0.700
Diabetes mellitus	49.6	(33.6-66.6)	
No diabetes m ellitus	74.0	(61.9-86.0)	0.032

To further investigate mortality over 10 years, Cox proportional hazards modelling was used, with time to death or last follow-up as the dependant variable, and a range of categorical and continuous variables included in the model (Table 4). The log-log plot is one way to assess graphically whether the assumption of proportional hazards was reasonable. For the assumption to hold then the log-log plot should show the separate lines as approximately parallel to each other.

**Table 4:** Multivariate Analysis: Cox Proportional Hazards Model including age, co-morbidity group, presence or absence of: diabetes, ischaemic heart disease, peripheral vascular disease, vascular disease, cancer or Chronic obstructive pulmonary disease

	**B**	**SE**	**P value**	**Hazard ratio**	**95% CI for Hazard ratio**	
					**Lower**	**Upper**
Age group (65 years)	-.780	.352	.027	.458	.230	.914
High versus low comorbidity	-.479	.373	.199	.619	.298	1.287
Diabetes	-.814	.363	.025	.443	.218	.902
Ischaemic heart disease	.235	.430	.585	1.265	.544	2.938
Cerebrovascular accident	-.421	.423	.319	.656	.287	1.502
Peripheral vascular disease	.399	.437	.361	1.491	.633	3.512
Vascular disease	-.568	.514	.269	.567	.207	1.553
Cancer	-.444	.404	.273	.642	.290	1.418
Chronic obstructive pulmonary disease	-.424	.651	.514	.654	.183	2.342

All statistical analyses were completed on SPSS for Windows (v19). A p-value of < 0.05 was considered statistically significant.

## Results

The cohort of patients was followed up for a median of 8.7 years [mean of 8.8 ± 0.3 years] from the date each patient commenced dialysis therapy in 2001/2002 and a median of 10.5 years [mean of 11.9 ± 4.2 years] from presentation to a nephrologist.

Baseline characteristics and the preferred mode of dialysis remained concordant with the original trial [12], consisting of a mean age of 63 ± 1 yrs and predominantly Caucasian ethnicity (97.9%: one Asian and one Chinese patient). Mortality was evaluated at a median of 126 months (mean of 146 ± 4.2 months) from presentation to the renal service.

Thirty percent (29/94) of patients had been transplanted during the follow-up period and were therefore censored in the survival cumulative analysis. At the time of last follow-up 18 of the transplant patients were still alive with a functioning renal allograft (62%); 2 had experienced transplant failure and returned to haemodialysis and 9 of those who received transplants died (31%; 6 cardiac deaths, 1 septicaemia, 1 calciphylaxis and 1 related to treatment withdrawal). For transplant patients, the median time from first presentation to death was 8.9 years [mean of 9.8 ± 5.1 years], with an average wait of 1.3 ± 0.4 years between first dialysis and transplant. Survival from transplantation using Kaplan-Meier showed a mean of 75 months (95% CI 57-92) and compared with non-transplantation 63 months (95% CI 51-75), however this was not statistically significant (p = 0.26) but subject to both bias in patient selection and small numbers. These patients were then censored from the data when examining mortality on further survival analysis.

The mortality rate at the end of the 10-year study period was 60% (56/94). Causes of death are described in Table 1 with cardiac related and infection accounting for the majority (57%). There was an increase in the percentage of deaths from treatment withdrawal (13%) and deaths from other causes (14%). The other categories included malignancy and deaths related to chronic renal failure whilst on dialysis.

Table 2 shows the comparison between survivors and non-survivors at 10 years of dialysis. Survivors had a lower mean age (53 years) compared to non-survivors (69 years). Chi-squared analysis of the ordinate variables compared vascular disease, diabetes, co-morbidity and haemoglobin stratified into categories <110 g/L and >110 g/L. At 10 years, those with vascular disease, diabetes and >3 co-morbidities were more likely to have a greater percentage of non-survivors. There was no difference in percentage survival between groups based on haemoglobin over 10 years. ANOVA analysis of continuous variables showed a difference between serum creatinine (μmol/L), albumin (g/L) and haemoglobin concentration (g/L) all at the start of RRT. Non-survivors at 10 years had a lower serum creatinine, lower albumin and haemoglobin at the start of RRT. There was no significant difference between triglycerides, calcium phosphate product, cholesterol, eGFR or serum creatinine at referral to a nephrologist or eGFR at commencement of RRT. Multivariate analysis using a Cox proportional hazards model showed a lack of statistical significance in this cohort, with the exception of age and diabetes (Table 4), the assumption of proportional hazards was verified by the log-log plots (Additional file 1).

**Figure 1:**
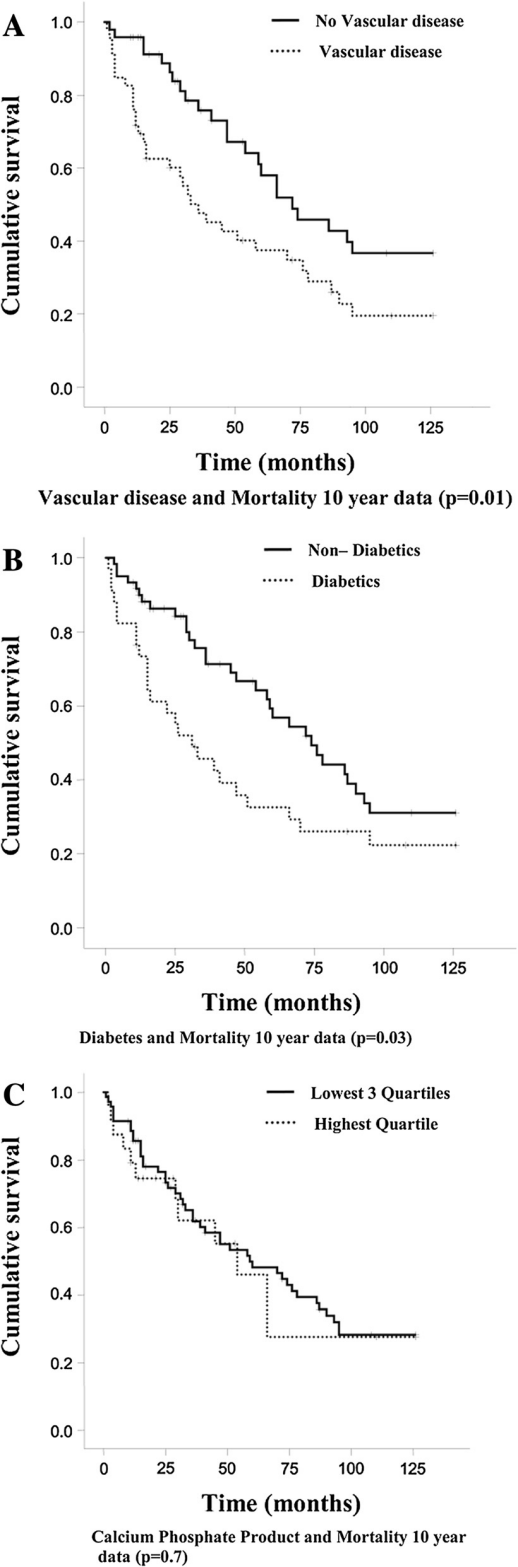
**Kaplain-Meier survival curves over 10 years for main risk factors. (A)** Vascular disease versus no vascular disease; **(B)** Diabetes versus no diabetes; **(C)** Calcium phosphate product quartiles.

On 10-year analysis, vascular disease and diabetes remained independent risk factors and were predicative of mortality (Table 3 and Additional file 1). From Kaplan-Meier survival curves, patients with vascular disease had a cumulative survival of 20% compared to 37% of those without vascular disease (Figure 1A; p = 0.01). Patients with diabetes had a cumulative survival of 22% compared to 31% without diabetes (Figure 1B; p = 0.03). Those aged greater than 65 years had a mean survival time of 51 months compared to those less than 65 years who had a mean survival of 85 months (p = 0.001). No significant difference was seen between those with the upper quartile of calcium phosphate product and the lowest 3 quartiles (Figure 1C; p = 0.7).

Additionally, patients stratified into high (>3) and low (<3) co-morbidities, unplanned or referred presentations and age groups stratified into greater and less than 65 years had marked differences in overall survival. Kaplan-Meier survival curves showed that those with high co-morbidities (>3) had a cumulative survival of 46% compared to 9% (p < 0.0001). Comparing clinical presentations, those with planned presentations had a cumulative survival of 43% compared to 12% of those with unplanned presentations (p = 0.004). Finally, those who were older (>65) had worse survival rates after 10 years of 15% compared to those in the younger age group, which showed just over half of these patients were still alive (52%) at 10 years (p = 0.001). Notably those who were aged over 65 represented 73% of the high co-morbidity cohort (30/41). Survival analysis of factors including LVH, duration of follow up, smoking status and type of dialysis and individual co-morbidities such as cancer were non-significant at 10 years (data not shown).

Risk calculation based on the risk calculator was predictive of stating dialysis. Patients stratified to low risk had a significantly longer time to dialysis; an average of 31 months (95% CI: 19.5-43.5) compared to high risk individuals who had an average time of 1 year (95% CI: 0.46-1.56). However patient mortality was not dependent on risk calculator categorisation.

**Figure 2:**
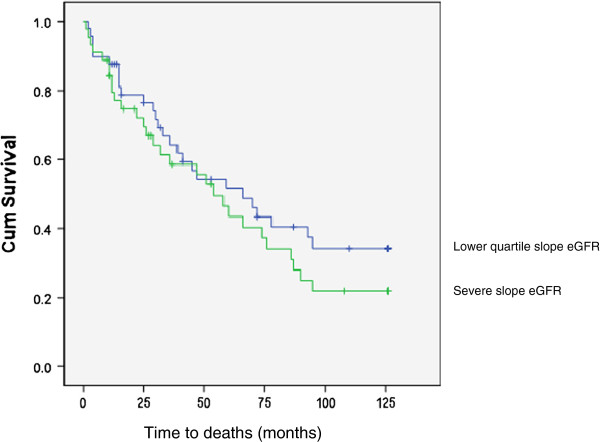
Kaplan-Meier Survival curves for predicting the mortality of those with severe slope eGFR deterioration (highest quartile – lower line) prior to commencement of dialysis compared to the lower 3 quartiles of rate of eGFR progression (upper line).

Kaplan-Meier survival analysis (Figure 2) showed that the severe slope eGFR group was associated with an increased mortality. The most severe slope deterioration in eGFR was generated at 52.8 months (95% CI: 38.5-67.1months) compared to 73.2 months (95% CI: 60.2-86.3-months) for the lower quartiles: p = 0.05) but this appeared to regress to the mean after 10 years.

## Discussion

In this single centre study of a relatively elderly Caucasian population receiving RTT, we have demonstrated that diabetes and vascular disease remain strong predictors of mortality, however contrary to the 5 year data a high calcium-phosphate product no longer correlates with mortality at 10 years. Vascular disease and sepsis account for the majority of causes of death at all stages of dialysis, accounting for 72% of the mortality seen at 5 years reducing to 57% at 10 years with a quarter of all mortality (14/56) represented by cardiac disease.

At 10 years, treatment withdrawal and other causes of death (including respiratory failure, pulmonary oedema and sudden death) collectively have shown the greatest proportional increase over the last 5 years (Table 1). Treatment withdrawal has become a more significant feature and is a common sequelae for those with end stage renal failure (ESRF) on long-term RRT. In some cohorts, treatment withdrawal has been described as an increasingly common cause of death and is related to an aging population with increased co-morbidities, such as diabetes, or occurs in young individuals with severe disease [16,17]. Indeed the poor projected survival, reduced added benefit of therapy and increased cognitive impairment seen in ESRF coupled with changing views of clinicians has increased dialysis withdrawal rates. Although we have investigated all-cause mortality as our primary outcome, it is important to note that other end-points for palliative care such as quality of life, ability to make advanced directives and symptom management are more clinically relevant.

The 5 year cumulative survival of patients with diabetes and non-diabetics was 47% vs. 67% (p = 0.03) and then at 10 years was of 22% vs. 31% respectively (Figure 1B; p = 0.03). At 5 years the paths of the two groups were divergent, however the 10 year graph is following a divergent-convergent path showing that therapy may be delaying the natural progression of the underlying disease or that those with diabetes may be experiencing early mortality.

The prevalence of diabetes mellitus in chronic kidney disease (CKD) continues to increase and remains a strong independent cardiovascular risk factor for patients in the general population as well as on dialysis [18]. Hyperglycaemia, in combination to traditional risk factors not only potentates endothelial damage and inflammation but in addition to diabetic mediators such as reactive oxygen species and cytokines, affects gene structure and function, generating targets for novel epigenetic therapeutics [19]. In addition to the recognised atherosclerotic processes responsible for these cardiac events, a decreased cardiac velocity reserve has also been observed in diabetic patients with CKD [20]. The importance of atherosclerosis in this cohort has been well documented and the presence of microalbuminuria has been recognised as a surrogate marker of endothelial injury and hence vascular damage seen in diabetic patients [21].

Intensive intervention of diabetes within a multidisciplinary team, in comparison to a conventional therapeutic approach, has been shown not only to reduce mortality from cardiovascular disease but also reduce the micro and macro-vascular complications of diabetes such as neuropathy, nephropathy and retinopathy [22,23].

Debate remains about the optimal level of glycaemic control for patients with diabetes on RRT [24]. Poor glycaemic control has been associated with increased cardiovascular deaths seen after transplantation [25]. In our study 67% of those transplanted suffered cardiac deaths (30% of whom were diabetic). Aggressive glycaemic control has been shown to be detrimental generating a U-shaped curve for HbA1c. When a patient’s HbA1c is persistently lowered below 6% this is associated with adverse outcomes, demonstrated not only in the general population, but in those receiving dialysis as publicised in the Action to Control Cardiovascular Risk in type 2 Diabetes Mellitus trial [26,27]. Although initially aimed at reducing microvascular complications, the increasing cardiovascular mortality that was seen meant the ACCORD trial had to be terminated prematurely. Although near-normal glycaemia has not been shown to reduce cardiovascular events in the short-term [28], indeed the majority of our cardiovascular disease mortality was seen within the first 5 years; follow up of long-term randomised control studies is needed to determine optimal glycaemic control. Indeed HbA1c at low levels do not necessarily only reflect diabetic treatment but can also be a reflection of malnourishment. Changes in body composition (e.g. muscle loss) may be associated with outcomes but was not examined.

Our data is in agreement to the data presented in previous studies demonstrating that patients receiving RRT with atherosclerotic vascular disease have higher mortality rates [29,30]. Also the number of risk factors for cardiovascular disease increases with the stage of CKD [31]. Given that the pre-dialysis diagnosis of cardiovascular disease predicted both early and late mortality, we speculate that the reemphasis of both prompt recognition and management of cardiovascular risk factors during the early stages of CKD maybe key to improving survival.

The importance of tight blood pressure regulation has been shown to significantly reduce mortality and morbidity [32]. However studies have raised concerns about over aggressive blood pressure reduction as blood pressure in relation to mortality, in a situation analogous to diabetes, also follows a U-shaped curve [33,34]. Statins have been used successfully in the general population to reduce cardiovascular events by around 34%. In CKD and dialysis patients the data is more complex. Initial studies have shown reduced LDL levels in dialysis patients with no cardiovascular benefit [35], while the more recent SHARP study has shown a 17% reduction in cardiovascular events in the overall cohort [36]. Sub-group analysis however suggests once again no benefit in dialysis patients [37]. Our analysis did not include blood pressure but instead used cardiac end organ markers of hypertension to indicate the presence of cardiac hypertrophy (ECG changes or cardiac echo). Direct relationship between levels of ambulatory blood pressure and cardiovascular events has not been clearly established by controlled studies in dialysis patients and there is a lack of a significant correlation between blood pressure and cardiovascular events in dialysis patients and no predictive value for mortality. It is also well recognised that blood pressure is subject to error in measurement and consistency and lack of standardization of various factors: the auscultatory method of blood pressure measurement eg Korotkoff sounds; patient position during blood pressure assessment; appropriate cuff size to ensure the cuff bladder encircles at least 80% of the arm is not always performed [38,39].

Traditional risk factors have been the mainstay of cardiovascular disease primary prevention identified in the Framingham Heart Study. However there is debate whether the same relative risks apply to a dialysis population in the secondary prevention of cardiovascular disease. The presence of “renal-specific” non-traditional risk factors including endothelial dysfunction, inflammation, oxidative stress, insulin resistance, anaemia and changes in vitamin D metabolism may play an even more important role in cardiovascular disease progression in CKD [40]. Therefore in considering the overall cardiovascular risk factors, a risk score specific to this population whilst incorporating a wide range of factors, including those prior to dialysis (such as renal function at the start of dialysis and microalbuminuria), may aid the stratification of high risk patients [41,42].

Application of the Tangri risk calculator was predictive of commencing RRT but not mortality while slope change in eGFR was more predictive of mortality suggesting rapid progressors to RRT are more likely to die. This would be in keeping with CKD stage in relation to cardiovascular risk [43] and potentially a future non-traditional risk factor to modify cardiovascular risk.

Although, in the previous analysis an elevated calcium phosphate product had a significant effect on early mortality and at 5years, validating previous findings in patients with ESRF [44], this was no longer the case after 10 years of follow up (Figure 1C; p = 0.7), reflecting other studies with a similar case mix and follow up [45]. One hypothesis is perhaps that calcium phosphate is an independent risk factor, or proxy, for those already with significant vascular disease. Those with higher proportions of vascular disease coupled with calcium and phosphate’s reflecting potential statistical predictors of cardiac calcification and mortality may have already perished at 1 and 5 years [46]. Indeed more aggressive management of vascular risk factors may account for the reduced mortality seen at 10 years. This is, however, speculation of an independent risk factor recorded on first dialysis and would require serial calcium phosphate measurements within a randomised control trial to validate this hypothesis.

There is emerging interest between vitamin D, FGF-23 and Klotho and their relation to cardiovascular risk in patients receiving RRT. An understanding how these factors modulate signalling pathways of mineral metabolism may be influential in modifying patients’ overall vascular risk [47,48]. A reduction in Klotho and elevation in FGF-23 culminate in reduced vitamin D levels and increased phosphate with subsequent increased risk of vascular calcification [49]. From our study perspective, levels of these signalling messengers may have changed with RRT or been influenced by the inherent genetic variations seen in different patient sub-groups altering expression of these factors and conferring protection - a concept proposed by those undertaking molecular biological assays with Klotho [50,51].

## Conclusions

Diabetes and vascular disease remain challenging risk factors in RRT patients. Vascular calcification in relation to a higher calcium-phosphate product is a potential and important modifiable risk factor to prevent early mortality on RRT for those patients already at significant cardiovascular risk. One might speculate that early aggressive intervention will be necessary to influence mortality in subsequent cohorts.

The burden of vascular disease remains of key importance for patients. Modifying the risk profile and intensive intervention accounts for the reduced numbers of cardiovascular events seen in the last 5 years of this study. It however seems more likely that the distribution of causes of death changes because those who die of cardiovascular or infectious causes die earlier. Determining modifiable risk factors and optimal management for those who are more likely to suffer from early demise will be central to changing the overall mortality.

### Limitations to the study

This study has several limitations. One acknowledges that this was an observational cohort study in a relatively elderly Caucasian group of patients and that small numbers were used. In particular a significant limitation was the use of risk factors taken from baseline only as it should be acknowledged that these factors may change over time and the associations may weaken with time. However it does highlight some interesting findings. Therefore prospective studies are necessary to investigate any potential causal relationships between both traditional and non-traditional risk factors that we have described with cardiovascular end-points. Larger numbers would be necessary to remove possible type 2 errors occurring and to see if interventions to reduce risk factors lead to overt reductions in cardiovascular risk. Other limitations include confounding by both known and unknown factors; misclassification of the outcome (cause of death only). Finally a weakness is the lack of longitudinal data on all the risk factors, biomarkers (such as Ca-P), vascular access, and comorbid conditions which may be less relevant closer to death or change during RRT.

### Short summary

Vascular and diabetic risk factors are strongly predictive of mortality in dialysis patients at 10 years, whereas calcium and phosphate are no longer. Treatment withdrawal has become a more salient feature. As patient’s risk profile changes over time, identifying modifiable risk factors at different stages of disease progression will help reduce morbidity and mortality of those on dialysis.

## Competing interests

The authors declare that they have no competing interests.

## Authors’ contributions

OTB carried out the updated data collection and initial analysis with VA, the statistician. He wrote the first draft of the paper. VA was responsible for all the statistical analysis, revision of the manuscript and subsequent analysis. SB collected the data set. He coordinated the study and hypothesis for the study. He reviewed the draft copy and revised the subsequent versions. All authors read and approved the final manuscript.

## Pre-publication history

The pre-publication history for this paper can be accessed here:

http://www.biomedcentral.com/1471-2369/15/20/prepub

## Supplementary Material

Additional file 1 **Table 3 analysis.** Assumption of Proportional Hazards. The hazards are consistent and do not vary differently over time. The following are the Log-Log plots. If PH model is true then the curves should be approximately parallel. **Figure S1.** Log_log plot for age. **Figure S2.** Log_log plot for vascular disease. **Figure S3.** Log_log plot for diabetes. **Figure S4.** Log_log plot for calcium phosphate product. Click here for file
