# Supplementary material for: Analysis of factors predicting mortality of new patients commencing renal replacement therapy 10 years of follow-up
Source: BMC Nephrol. 2014 Jan 20;15:20. doi: 10.1186/1471-2369-15-20 (PMC3902419; doi:10.1186/1471-2369-15-20)

Additional file 1: Table 3 analysis. Assumption of Proportional Hazards

The hazards are consistent and do not vary differently over time. The following are the Log-Log plots. If PH model is true then the curves should be approximately parallel.

Figure S1: Log_log plot for age


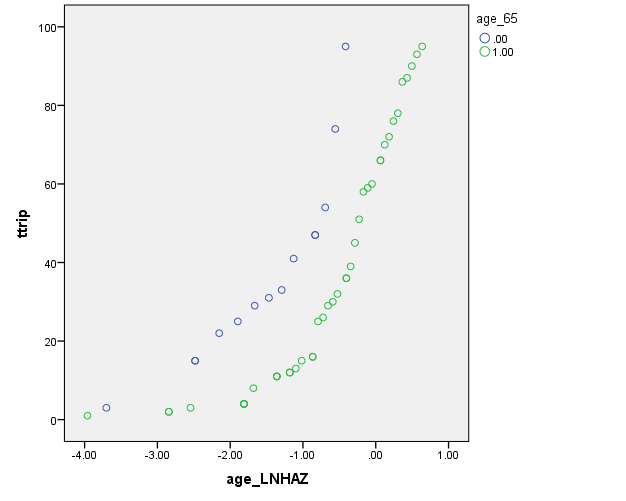


Figure S2: Log_log plot for vascular disease


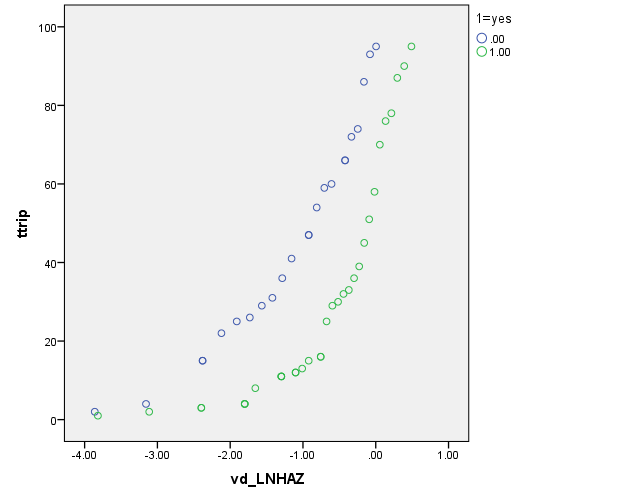


Figure S3: Log_log plot for diabetes


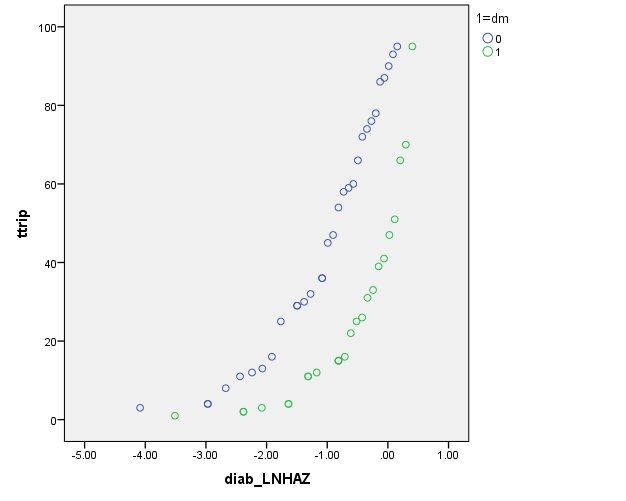


Figure S4: Log_log plot for calcium phosphate product


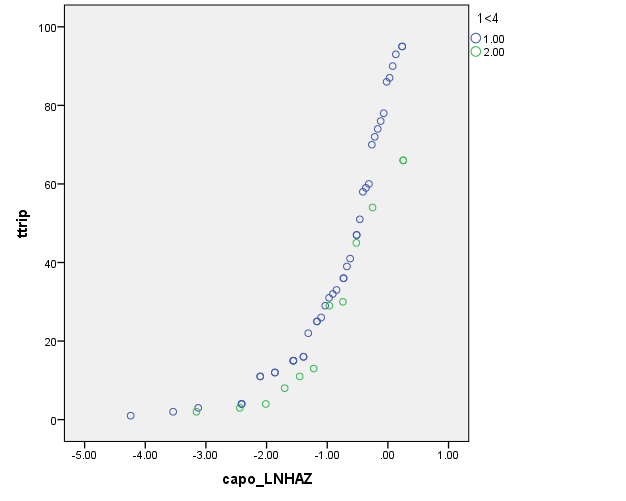

Supplement: Additional file 1 — Table 3 analysis. Assumption of Proportional Hazards. The hazards are consistent and do not vary differently over time. The following are the Log-Log plots. If PH model is true then the curves should be approximately parallel. Figure S1. Log_log plot for age. Figure S2. Log_log plot for vascular disease. Figure S3. Log_log plot for diabetes. Figure S4. Log_log plot for calcium phosphate product. [file 1471-2369-15-20-S1.docx]
